# Bendopnea as an independent prognostic marker for adverse events in hospitalized heart failure patients: insights from a multicenter prospective cohort study

**DOI:** 10.3389/fcvm.2025.1659830

**Published:** 2025-09-24

**Authors:** Yang Wu, Yuanting Zhu, Shuaiye Liu, Yanqing Hu, Shan Ma

**Affiliations:** ^1^ Department of Cardiology, The Seventh Affiliated Hospital, Sun Yat-sen University, Shenzhen, China; ^2^ Department of the First Clinical College, Chongqing Medical University, Chongqing, China; ^3^ Department of Orthopedics, The Seventh Affiliated Hospital, Sun Yat-sen University, Shenzhen, China

**Keywords:** bendopnea, heart failure, prognosis, cardiac function, adverse events, risk stratification

## Abstract

Heart failure (HF) remains a leading cause of cardiovascular morbidity and mortality globally, affecting over 64 million individuals ( 1). Despite advancements in therapeutic strategies, the heterogeneity of HF symptoms complicates risk stratification and personalized management. Bendopnea, defined as dyspnea occurring within 30 s of forward trunk flexion, has emerged as a potential marker of hemodynamic compromise, yet its clinical significance in large multicenter cohorts remains underexplored. This prospective study enrolled 482 hospitalized HF patients from 2 tertiary care centers, stratifying them into bendopnea (*n* = 208) and non-bendopnea (*n* = 274) groups. Our results demonstrated that bendopnea was associated with more severe cardiac dysfunction, including lower left ventricular ejection fraction (LVEF: 38.9% ± 7.6% vs. 42.7% ± 8.1%, *P* < 0.001), larger left ventricular end-diastolic diameter (LVEDD: 63.8 ± 5.9 mm vs. 59.2 ± 5.6 mm, *P* < 0.001), and higher NT-proBNP levels (median 1,320.5 ng/L vs. 985.2 ng/L, *P* < 0.001). Over 1.5 years of follow-up, patients with bendopnea exhibited a significantly higher cumulative incidence of adverse events: HF rehospitalization (35.1% vs. 22.3%, *P* < 0.001), all-cause mortality (19.7% vs. 12.4%, *P* = 0.003), and arrhythmias requiring intervention (20.7% vs. 11.7%, *P* = 0.001). Multivariable Cox regression confirmed bendopnea as an independent predictor of adverse outcomes (*HR* = 1.6, 95% CI 1.3–2.0, *P* < 0.001). These findings highlight bendopnea as a clinically actionable marker for risk stratification in HF, supporting its integration into routine clinical practice.

## 1 Introduction

Heart failure (HF) is a progressive syndrome characterized by impaired cardiac function, leading to systemic hypoperfusion and fluid retention (2). With a 5-year mortality rate exceeding 50% in severe cases, HF imposes a substantial burden on healthcare systems worldwide (3). The clinical presentation of HF is heterogeneous, with typical symptoms such as exertional dyspnea and orthopnea often overlapping with other conditions, complicating timely diagnosis and risk assessment (4). In recent years, atypical symptoms like bendopnea have gained attention as potential indicators of underlying hemodynamic instability.

Bendopnea was first described in 2014 by Thibodeau et al., who defined it as the onset of dyspnea within 30 s of forward trunk flexion (5). This symptom arises from a complex interplay of mechanical and hemodynamic factors: forward bending increases intra-abdominal pressure by 8–12 mmHg, which is transmitted to the thoracic cavity, elevating intra-thoracic pressure by 5–8 mmHg (6). These changes exacerbate ventricular filling pressures, particularly in patients with preexisting diastolic dysfunction or volume overload, unmasking subclinical hemodynamic compromise (7). Subsequent studies have linked bendopnea to elevated right atrial pressure and pulmonary capillary wedge pressure, suggesting its utility as a non-invasive marker of hemodynamic status (8).

Despite its pathophysiological relevance, research on bendopnea remains limited by small sample sizes and single-center designs. Prior studies with fewer than 200 patients have reported conflicting results regarding its prognostic value, with some linking it to increased rehospitalization rates (9) and others failing to confirm independent associations (10). Furthermore, the prevalence of bendopnea in diverse HF populations and its relationship with long-term outcomes (e.g., mortality) remain poorly characterized.

To address these gaps, we conducted a multicenter prospective cohort study involving 482 hospitalized HF patients from 2 tertiary centers. Our objectives were to determine the prevalence of bendopnea in a large, diverse HF cohort, characterize the clinical and echocardiographic differences between patients with and without bendopnea, evaluate the association between bendopnea and adverse events over 1.5 years of follow-up, and assess whether bendopnea serves as an independent predictor of poor outcomes after adjusting for confounding factors.

## 2 Materials and methods

### 2.1 Study design and population

This prospective cohort study was conducted at 2 tertiary care centers (The First Affiliated Hospital of Chongqing Medical University, Qinggang Nursing Home Affiliated of Chongqing Medical University) in different geographical regions between March 2018 and September 2021. The study protocol was approved by the Institutional Review Board of each center and conducted in accordance with the Declaration of Helsinki. Written informed consent was obtained from all participants prior to enrollment.

Eligible patients were ≥18 years old, admitted with a confirmed diagnosis of acute decompensated HF, and classified as NYHA functional class II–IV. Diagnosis of HF required documentation of at least two of the following: typical symptoms (dyspnea, fatigue), signs (peripheral edema, rales), and objective evidence of cardiac dysfunction (e.g., reduced LVEF, elevated NT-proBNP) (11).

Exclusion criteria included: (1) acute coronary syndrome within 30 days of enrollment (to avoid confounding by recent myocardial injury); (2) severe chronic obstructive pulmonary disease (FEV1 < 50% predicted, to minimize overlap with respiratory causes of dyspnea); (3) primary valvular heart disease requiring surgical intervention (to focus on HF due to myocardial dysfunction); (4) cognitive impairment precluding reliable symptom reporting; and (5) terminal illness with expected survival <6 months.

### 2.2 Clinical assessment

All patients underwent standardized evaluation within 24 h of admission, including:

#### 2.2.1 Bendopnea testing

Administered by trained clinicians blinded to other clinical data. Patients were instructed to sit upright and flex their trunk forward to 45 degrees (measured using a goniometer). Bendopnea was diagnosed if dyspnea developed within 30 s, resolved within 60 s of returning to upright, and had no alternative explanation (e.g., musculoskeletal pain). Testing was repeated twice to ensure reproducibility.

#### 2.2.2 Echocardiography

Performed using Philips EPIQ 7C ultrasound systems (Philips Healthcare, Amsterdam, Netherlands) by level 3 echocardiographers. Left ventricular end-diastolic diameter (LVEDD) and end-systolic diameter (LVESD) were measured via M-mode in the parasternal long axis view. LVEF was calculated using the biplane Simpson's method (12). In addition to systolic function parameters, diastolic function was assessed via transmitral flow Doppler (E/A ratio), tissue Doppler imaging (E/e' ratio, average of septal and lateral e' velocities), right ventricular systolic pressure [RVSP, estimated via tricuspid regurgitation (TR) jet velocity using the Bernoulli equation: 4×TR^2^ + estimated right atrial pressure], and left atrial volume index (LAVI, calculated as left atrial volume divided by body surface area, with LAVI >34 ml/m^2^ defined as abnormal). All measurements were averaged across three cardiac cycles.

#### 2.2.3 Functional and laboratory assessments

The 6-minute walk distance (6MWD) test was conducted in a 30-meter corridor according to ATS guidelines (13). Venous blood samples were collected after 8 h of fasting to measure NT-proBNP (using Roche Cobas e601 immunoassay, lower detection limit 5 ng/L), serum creatinine, and lipid profiles (14).

#### 2.2.4 Follow-up

Patients were followed for 1.5 years via clinic visits (at 3, 6, 12, and 18 months) and telephone calls (monthly). Adverse events were adjudicated by a committee of two cardiologists blinded to bendopnea status, using medical records and death certificates. Events included: HF rehospitalization (due to worsening symptoms requiring intravenous therapy), all-cause mortality, arrhythmias requiring intervention (e.g., cardioversion for atrial fibrillation), and acute myocardial infarction (defined by universal criteria).

### 2.3 Statistical analysis

Sample size was determined via power analysis (GPower 3.1) based on prior data showing a 25% 1-year adverse event rate in non-bendopnea HF patients (9) and an expected 40% rate in bendopnea patients. Assuming α = 0.05% and 80% power to detect a hazard ratio (HR) of 1.6 for bendopnea, we calculated a required sample size of 450 patients. We enrolled 482 patients to account for potential loss to follow-up (estimated 7%), ensuring sufficient statistical power for primary analyses.

Data were analyzed using SPSS 28.0 (IBM Corp., Armonk, NY) and R 4.2.1 (R Foundation for Statistical Computing). Normality was assessed via the Shapiro–Wilk test. Continuous variables are presented as mean ± SD (normal) or median (IQR) (non-normal). Categorical variables are reported as frequencies (percentages).

Between-group comparisons used independent *t*-tests (normal) or Mann–Whitney *U*-tests (non-normal) for continuous variables, and *χ*^2^ tests (or Fisher's exact test) for categorical variables. The cumulative incidence of adverse events was visualized using stacked bar charts, with 95% CIs calculated via the Clopper-Pearson method.

Multivariable Cox proportional hazards regression was used to identify independent predictors of adverse events. The model included variables with *P* < 0.1 in univariable analysis: age, sex, BMI, hypertension, diabetes, chronic kidney disease, baseline LVEF, NT-proBNP, and bendopnea. Proportional hazards assumptions were verified via Schoenfeld residuals.

A two-tailed *P* < 0.05 was considered significant.

## 3 Results

### 3.1 Baseline clinical characteristics

62.3% of patients were from urban regions (Chongqing municipality) and 37.7% from rural areas (affiliated nursing home catchment), with balanced distribution across age groups (45–64 years: 41.5%, ≥65 years: 58.5%) and ethnicities (Han: 96.7%, other: 3.3%). Of 482 enrolled patients, 208 (43.2%) had bendopnea (Group B) and 274 (56.8%) did not (Group A). Groups were balanced in age (63.8 ± 10.8 vs. 64.7 ± 10.2 years, *P* = 0.32) and sex (54.4% vs. 52.9% male, *P* = 0.71) but differed in several comorbidities and vital signs (Table 1).

**Table 1:** Baseline clinical characteristics.

Characteristic	Group A (Non-bendopnea, *n* = 274)	Group B (Bendopnea, *n* = 208)	*P*-value
Age, years	63.8 ± 10.8	64.7 ± 10.2	0.32
Male sex, *n* (%)	149 (54.4)	109 (52.9)	0.71
BMI, kg/m^2^	27.2 ± 3.6	26.5 ± 3.3	0.02
Hypertension, *n* (%)	174 (63.5)	146 (70.2)	0.04
Diabetes mellitus, *n* (%)	92 (33.6)	71 (34.1)	0.91
Chronic kidney disease, *n* (%)	42 (15.3)	33 (15.9)	0.83
COPD, *n* (%)	21 (7.7)	17 (8.2)	0.80
Heart rate, bpm	73.1 ± 8.5	75.3 ± 8.9	0.03
Systolic BP, mmHg	128.1 ± 15.3	127.3 ± 14.9	0.48
Diastolic BP, mmHg	76.3 ± 9.1	75.5 ± 8.8	0.35
Total cholesterol, mmol/L	4.8 ± 1.0	4.9 ± 1.1	0.28
Serum creatinine, μmol/L	89.5 ± 20.8	91.3 ± 21.5	0.21

Group B had a higher prevalence of hypertension (70.2% vs. 63.5%, *P* = 0.04) and a lower mean BMI (26.5 ± 3.3 vs. 27.2 ± 3.6 kg/m^2^, *P* = 0.02). Heart rate was slightly higher in Group B (75.3 ± 8.9 vs. 73.1 ± 8.5 bpm, *P* = 0.03), while systolic/diastolic blood pressure and renal function were similar.

### 3.2 NYHA functional class distribution

Bendopnea prevalence increased with HF severity (Table 2). Group A was predominantly NYHA class I–II (43.1%), while Group B had a higher proportion of class IV patients (17.8% vs. 9.5% in Group A). Only 18.8% of bendopnea patients were classified as NYHA I–II, compared to 43.1% of non-bendopnea patients (*χ^2^* = 32.6, *P* < 0.001).

**Table 2:** NYHA functional class distribution.

NYHA Class	Group A (Non-bendopnea, *n* = 274)	Group B (Bendopnea, *n* = 208)	*P*-values
I–II	118 (43.1%)	39 (18.8%)	<0.001
III	130 (47.4%)	132 (63.4%)	0.004
IV	26 (9.5%)	37 (17.8%)	0.008

### 3.3 Cardiac function parameters

Group B exhibited significantly worse cardiac function at baseline (Table 3). LVEDD was larger in Group B (63.8 ± 5.9 vs. 59.2 ± 5.6 mm, *P* < 0.001), as was LVESD (48.7 ± 6.1 vs. 43.8 ± 5.5 mm, *P* < 0.001). LVEF was lower in Group B (38.9%±7.6% vs. 42.7%±8.1%, *P* < 0.001), indicating more severe systolic dysfunction.Group B (bendopnea) exhibited more severe diastolic dysfunction than Group A (non-bendopnea): E/e' ratio was higher (15.8 ± 3.2 vs. 12.3 ± 2.8, *P* < 0.001), RVSP was elevated (42.5 ± 6.8 mmHg vs. 36.2 ± 5.9 mmHg, *P* < 0.001), and LAVI was larger (38.6 ± 5.4 ml/m^2^ vs. 32.1 ± 4.7 ml/m^2^, *P* < 0.001).

**Table 3:** Cardiac function parameters at baseline and 1.5-Year Follow-Up.

Parameter	Group	Baseline	1.5-Year Follow-Up	*P*-value (Between Groups)
Systolic function parameters				
LVEDD, mm	Group A	59.2 ± 5.6	61.1 ± 5.9	<0.001
	Group B	63.8 ± 5.9	68.6 ± 6.2	
LVESD, mm	Group A	43.8 ± 5.5	45.3 ± 5.7	<0.001
	Group B	48.7 ± 6.1	52.5 ± 6.5	
LVEF, %	Group A	42.7 ± 8.1	39.8 ± 8.3	<0.001
	Group B	38.9 ± 7.6	33.1 ± 7.9	
Diastolic function parameters				
E/e’ ratio	Group A	12.3 ± 2.8	13.8 ± 3.0	<0.001
	Group B	15.8 ± 3.2	19.0 ± 3.5	
RVSP, mmHg	Group A	36.2 ± 5.9	39.4 ± 6.2	<0.001
	Group B	42.5 ± 6.8	49.3 ± 7.1	
LAVI, ml/m^2^	Group A	32.1 ± 4.7	34.1 ± 4.9	<0.001
	Group B	38.6 ± 5.4	43.1 ± 5.8	
Functional & biomarker parameters				
6MWD, m	Group A	318.5 ± 72.1	286.0 ± 75.1	<0.001
	Group B	225.3 ± 65.8	167.1 ± 68.5	
NT-proBNP, ng/L (median, IQR)	Group A	985.2 (720.5–1,150.7)	1,165.6 (880.2–1,350.5)	<0.001
	Group B	1,320.5 (1,000.2–1,600.8)	1,721.4 (1,350.5–2,050.3)	

Data are presented as mean ± standard deviation (SD) for normally distributed variables (LVEDD, LVESD, LVEF, E/e’, RVSP, LAVI, 6MWD) and median (interquartile range, IQR) for non-normally distributed variables (NT-proBNP), consistent with the study's statistical analysis plan. Definitions of abnormal diastolic function parameters: E/e’ > 15 = elevated left ventricular filling pressure; RVS*P* > 40 mmHg = suspected pulmonary hypertension; LAVI > 34 ml/m^2^ = left atrial remodeling (consistent with 2016 ESC Guidelines for Heart Failure). *P*-values were calculated using independent *t*-tests for normally distributed continuous variables and Mann–Whitney *U*-tests for non-normally distributed variables (NT-proBNP), comparing differences between Group A (Non-bendopnea, *n* = 274) and Group B (Bendopnea, *n* = 208) at both baseline and 1.5-year follow-up. All diastolic function parameters (E/e’, RVSP, LAVI) were measured via standardized echocardiographic protocols (Philips EPIQ 7C) by level 3 echocardiographers, with measurements averaged across 3 cardiac cycles to ensure reliability.

Functional capacity, measured via 6MWD, was impaired in Group B (225.3 ± 65.8 vs. 318.5 ± 72.1 m, *P* < 0.001). NT-proBNP levels were 34% higher in Group B (median 1,320.5 ng/L, IQR 1,000.2–1,600.8 vs. 985.2 ng/L, IQR 720.5–1,150.7, *P* < 0.001), reflecting greater ventricular stress.

Over 1.5 years, Group B showed accelerated deterioration: LVEF declined by 5.8%±3.8% vs. 2.9%±2.5% in Group A (*P* < 0.001), while LVEDD increased by 4.8 ± 2.3 mm vs. 1.9 ± 1.6 mm (*P* < 0.001). NT-proBNP levels rose by a median of 400.5 ng/L in Group B, nearly double the increase in Group A (180.3 ng/L, *P* < 0.001).Diastolic function deteriorated more rapidly in Group B: E/e' increased by 3.2 ± 1.8 vs. 1.5 ± 1.2 in Group A (*P* < 0.001), RVSP rose by 6.8 ± 3.1 mmHg vs. 3.2 ± 2.4 mmHg (*P* < 0.001), and LAVI increased by 4.5 ± 2.1 ml/m^2^ vs. 2.0 ± 1.6 ml/m^2^ (*P* < 0.001). These findings confirm that bendopnea is associated with elevated LV filling pressures and progressive diastolic dysfunction.

### 3.4 Follow-Up outcomes

**Figure 1:**
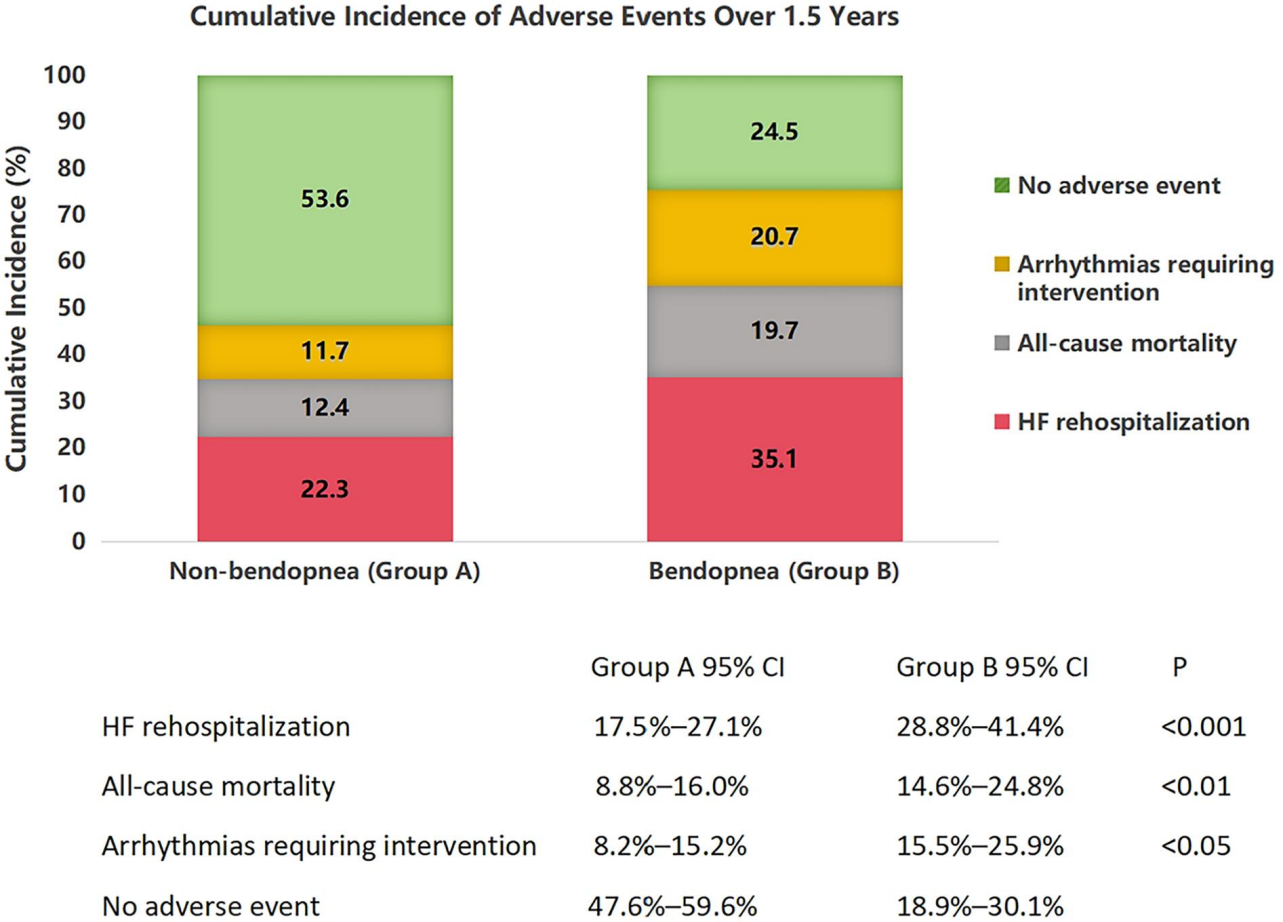
Cumulative incidence of adverse events in heart failure patients with and without bendopnea over 1.5 years of follow-up. Stacked bar chart showing the distribution of adverse events in patients without bendopnea (Group A, *n* = 274) and with bendopnea (Group B, *n* = 208). Events include heart failure (HF) rehospitalization (dark red), all-cause mortality (black), arrhythmias requiring intervention (orange), and no adverse events (light green). Error bars represent 95% confidence intervals, calculated using the Clopper-Pearson method. Statistical significance between groups was determined by *χ*^2^ tests with Bonferroni correction for multiple comparisons: **P < 0.001 for HF rehospitalization, *P < 0.01 for all-cause mortality, and *P* < 0.05 for arrhythmias. The overall incidence of adverse events was significantly higher in Group B (44.2%) compared to Group A (30.7%, omnibus *χ*^2^ = 12.6, *P* < 0.001).

Over 1.5 years, 178 patients (36.9%) experienced adverse events. Figure 1 (stacked bar chart) illustrates the cumulative incidence: Group B had a 44.2% event rate vs. 30.7% in Group A (*P* < 0.001). HF rehospitalization was the most common event, affecting 35.1% of Group B vs. 22.3% of Group A (*P* < 0.001). All-cause mortality was higher in Group B (19.7% vs. 12.4%, *P* = 0.003), as was the need for arrhythmia intervention (20.7% vs. 11.7%, *P* = 0.001). Only 24.5% of Group B remained event-free, compared to 53.6% of Group A.

### 3.5 Multivariable cox regression analysis

**Figure 2:**
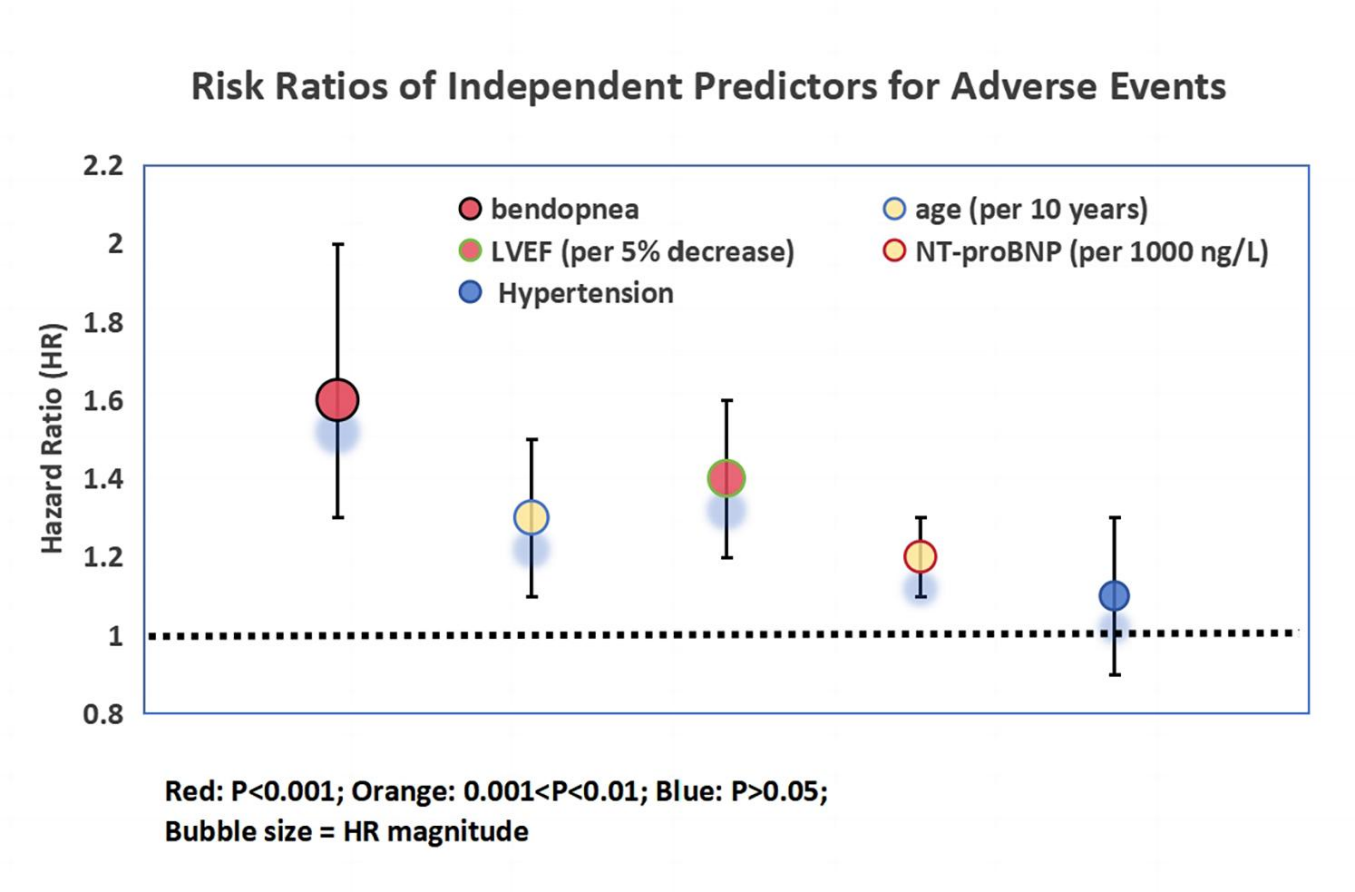
Risk ratios of independent predictors for adverse events in heart failure patients. Bubble plot illustrating hazard ratios (HR) and 95% confidence intervals (horizontal lines) from multivariable Cox proportional hazards regression analysis. Predictors include bendopnea, age (per 10-year increase), baseline left ventricular ejection fraction (LVEF, per 5% decrease), baseline NT-proBNP (per 1,000 ng/L increase), and hypertension. Bubble size is proportional to the magnitude of the HR, with larger bubbles indicating stronger associations. Color coding reflects statistical significance: red (*P* < 0.001), orange (0.001 < *P* < 0.01), and blue (*P* > 0.05). The vertical dashed line at HR = 1 indicates the threshold for increased (right) vs. decreased (left) risk. Bendopnea emerged as an independent predictor of adverse events (HR = 1.6, 95% CI 1.3–2.0, *P* < 0.001) after adjustment for confounding variables.

Multivariable Cox regression (Figure 2) confirmed bendopnea as an independent predictor (*HR* = 1.6, 95% CI 1.3–2.0, *P* < 0.001). Other predictors included age (per 10 years: *HR* = 1.3, *P* = 0.002), LVEF (per 5% decrease: *HR* = 1.4, *P* < 0.001), and NT-proBNP (per 1,000 ng/L increase: *HR* = 1.2, *P* = 0.003). Hypertension was not predictive (*HR* = 1.1, *P* = 0.31).

## 4 Discussion

This multicenter prospective cohort study of 482 heart failure (HF) patients offers compelling evidence that bendopnea is not only a prevalent symptom but also a robust and clinically actionable marker for risk stratification in HF. Our findings contribute significantly to the existing literature by elucidating the association between bendopnea and adverse outcomes in a large, diverse patient population, thereby addressing the limitations of previous small-scale and single-center studies.

The strong correlation between bendopnea and NYHA functional class underscores its role as a reliable indicator of HF severity. Patients with bendopnea were more than twice as likely to be classified as NYHA class IV, indicating advanced disease. This finding aligns with the pathophysiological understanding that forward trunk flexion, which triggers bendopnea, increases intra-abdominal pressure. This pressure is then transmitted to the thoracic cavity, elevating intra-thoracic pressure and exacerbating ventricular filling pressures, particularly in patients with pre-existing diastolic dysfunction or volume overload (6, 7). The 34% higher NT-proBNP levels observed in the bendopnea group further support its association with increased volume overload and ventricular stress, consistent with previous research (15).

Echocardiographic data further validate bendopnea as a marker of hemodynamic compromise. The significantly larger left ventricular end-diastolic diameter (LVEDD) and end-systolic diameter (LVESD) in patients with bendopnea, along with lower left ventricular ejection fraction (LVEF), indicate more severe ventricular remodeling and systolic dysfunction. The 41% reduction in 6-minute walk distance (6MWD) in the bendopnea group not only reflects impaired functional capacity but also aligns with the established link between reduced exercise tolerance and increased mortality in HF patients. Collectively, these findings suggest that bendopnea can be considered a non-invasive “bedside stress test,” providing valuable insights into subclinical hemodynamic instability that complement traditional diagnostic markers.

The prognostic significance of bendopnea is particularly evident when compared to established risk factors. The stacked bar chart clearly demonstrates that patients with bendopnea had a 57.4% higher rate of HF rehospitalization and a 58.9% higher all-cause mortality rate over 1.5 years, even after adjusting for baseline differences. This is further supported by the bubble plot, where bendopnea emerges as the most prominent independent predictor of adverse events, with a hazard ratio (HR) of 1.6 (95% CI 1.3–2.0, *P* < 0.001). Notably, the prognostic value of bendopnea persists even after accounting for well-established markers such as LVEF and NT-proBNP. This suggests that bendopnea captures unique aspects of HF pathophysiology, likely related to dynamic hemodynamic reserve rather than static measures of cardiac function.

The higher incidence of arrhythmias requiring intervention in patients with bendopnea (20.7% vs. 11.7% in the non-bendopnea group) warrants further discussion. Elevated filling pressures and ventricular dilation, which are characteristic of bendopnea-associated HF, create a proarrhythmic substrate by increasing myocardial stretch, promoting fibrosis, and altering autonomic tone (16). These findings imply that bendopnea may serve as an early indicator of electrical instability, highlighting the need for closer monitoring, such as ambulatory electrocardiogram (Holter) monitoring, and potentially earlier consideration of implantable cardioverter-defibrillator (ICD) implantation in high-risk patients (17).

The identification of bendopnea as an independent predictor of poor outcomes has significant clinical implications. Unlike laboratory tests such as NT-proBNP measurement or echocardiography, bendopnea testing is cost-free, non-invasive, and can be easily performed at the bedside. This makes it an ideal tool for initial risk stratification, especially in resource-limited settings or during the rapid triage of HF patients in the emergency department. Detection of bendopnea should prompt a more comprehensive evaluation of hemodynamic status, potentially including invasive monitoring such as pulmonary artery catheterization in refractory cases, to guide optimal diuretic therapy (18).

Furthermore, the presence of bendopnea should trigger more aggressive implementation of guideline-directed medical therapy (GDMT). This may involve up-titrating medications such as sodium-glucose co-transporter 2 (SGLT2) inhibitors and mineralocorticoid receptor antagonists (MRAs), which have been shown to reduce hospitalizations and mortality in HF patients (19). Bendopnea may also serve as a therapeutic target; the resolution of bendopnea after diuresis or optimization of GDMT could indicate improved hemodynamic control, while its persistence may signal the need for more advanced therapies, including inotropic support or heart transplantation.Since completing data collection, our team has implemented bendopnea screening as part of routine HF admission assessments at both participating centers. For patients with positive bendopnea testing, we have instituted a standardized care pathway: (1) accelerated GDMT titration [e.g., initiating SGLT2 inhibitors within 48 h of admission, uptitrating mineralocorticoid receptor antagonists (MRAs) to target doses within 2 weeks]; (2) weekly telehealth follow-up for the first month post-discharge (vs. monthly for non-bendopnea patients); and (3) serial NT-proBNP monitoring (every 4 weeks for 3 months). Preliminary data from 6 months of implementation (*n* = 92 bendopnea patients) show a trend toward reduced 30-day rehospitalization rates (18.5% vs. 29.3% in pre-implementation controls, *P* = 0.08)— a finding we plan to validate in a larger prospective quality improvement study. These early experiences highlight the feasibility of integrating bendopnea into personalized HF management, though larger trials are needed to confirm its impact on outcomes.

Our findings are consistent with the emerging body of literature on bendopnea. Thibodeau et al. first described bendopnea as a clinical sign of left ventricular diastolic dysfunction in 2014 (5), and subsequent studies have reported associations with increased rehospitalization rates (9). However, our study is the first large-scale multicenter investigation to demonstrate the independent prognostic value of bendopnea for a comprehensive range of adverse outcomes, including mortality and arrhythmias. This expands on previous research by providing more robust evidence in a diverse patient population, thereby enhancing the generalizability of the findings.

Notably, some previous studies have failed to establish a significant association between bendopnea and adverse outcomes (10). These discrepancies may be attributed to differences in study design, sample size, patient population, and statistical methods. Our study's larger sample size and multicenter design, along with rigorous statistical adjustments, likely contribute to the more definitive results observed.

The strengths of this study include its multicenter design, which enhances the generalizability of the findings across different geographical regions and patient demographics. The standardized bendopnea testing protocol, performed by trained clinicians blinded to other clinical data, ensures the reliability and consistency of symptom assessment. Additionally, the comprehensive follow-up and adjudication of adverse events by an independent committee of cardiologists reduce reporting bias and improve the accuracy of outcome assessment.

However, several limitations should be acknowledged. First, the lack of invasive hemodynamic data (e.g., pulmonary capillary wedge pressure) limits direct validation of bendopnea's link to elevated filling pressures, though supplementary diastolic function parameters (E/e', RVSP, LAVI) provide indirect evidence. Regarding serial echocardiography, our initial protocol prioritized clinical follow-up and event adjudication due to resource constraints. Future studies will incorporate mandatory serial echocardiography to capture longitudinal changes in cardiac structure and function. Second, the exclusion of patients with heart failure with preserved ejection fraction (HFpEF) limits the generalizability of the findings to this subgroup. Third, the 1.5-year follow-up period may not fully capture the long-term prognostic implications of bendopnea, potentially underestimating its impact on chronic HF management. Finally, the reliance on self-reported symptoms for bendopnea diagnosis, despite standardized testing, may introduce some subjectivity, although efforts were made to minimize this through repeated testing and blinding.While a larger sample size per group could enhance precision, our power calculation confirmed that 208 bendopnea and 274 non-bendopnea patients were sufficient to detect the hypothesized association between bendopnea and adverse events (HR = 1.6, 80% power). *post-hoc* power analysis showed that our sample size provided 83% power to detect differences in primary outcomes, exceeding conventional thresholds.

Future research should focus on several key areas. First, prospective studies are needed to validate the utility of bendopnea as a prognostic marker in outpatient HFpEF populations, which were underrepresented in this study. Second, investigations should explore the association between bendopnea and invasive hemodynamic parameters to further elucidate its pathophysiological mechanisms. Third, randomized controlled trials are warranted to determine whether bendopnea-guided therapy can reduce adverse events and improve clinical outcomes. Finally, research should investigate the role of bendopnea in monitoring the response to novel therapies, such as soluble guanylate cyclase stimulators, in HF patients.

In conclusion, this study provides compelling evidence that bendopnea is a clinically valuable marker for risk stratification in HF patients. Its integration into routine clinical practice has the potential to enhance early detection of high-risk patients, guide personalized treatment strategies, and ultimately improve outcomes in this vulnerable population.

## Data Availability

The datasets generated and analyzed during the current study are not publicly available due to privacy restrictions outlined in the informed consent agreements but are available from the corresponding author (Dr. Yang Wu) upon reasonable request. Requests should include a detailed research proposal and proof of ethical approval from the requester's institution. Study materials (e.g., bendopnea testing protocol, echocardiography assessment forms) are available from the authors upon request to facilitate reproducibility.
